# Difficult Management of Giant Gallstone Ileus of a Post-cardiopulmonary Resuscitation Patient: A Case Report

**DOI:** 10.7759/cureus.23911

**Published:** 2022-04-07

**Authors:** Mahmut Kaan Demircioğlu, Zeynep Gül Demircioğlu, Canver Önal, Sinem Özler

**Affiliations:** 1 General Surgery, Kars Harakani State Hospital, Kars, TUR; 2 Radiology, Kars Harakani State Hospital, Kars, TUR; 3 Anesthesiology, Kars Harakani State Hospital, Kars, TUR

**Keywords:** gastrointestinal obstruction, gallstone, enterotomy, emergency surgery, gallstone ileus

## Abstract

Gallstone ileus is a rare form of mechanical intestinal obstruction. It is associated with high mortality and morbidity in the elderly population. The treatment of gallstone ileus includes more than one surgical option and it is appropriate to determine the surgical technique according to the general condition of the patient.

In our case report, we present an 83-year-old patient who was admitted to the emergency room due to nausea and vomiting, was diagnosed with gallstone ileus, and had a cardiac arrest just before surgery. The patient management and the following surgical approach are also explained in detail.

## Introduction

Gallstone ileus is a rare etiology of mechanical intestinal obstruction, especially seen in the elderly population. It is seen in only 1-4% of patients who are hospitalized due to mechanical intestinal obstruction. The diagnosis is more difficult than the other causes of mechanical intestinal obstruction. It has high mortality and morbidity rates due to both being seen in elderly patients and other complications [1].

It has long been stated in the literature that early recognition of gallstone ileus reduces mortality [2]. The gold standard treatment for gallstone ileus is surgery. Gallstones must be removed by enterotomy during the surgery. While cholecystectomy and repair of cholecystoduodenal fistula can be performed simultaneously, it is also appropriate to remove only the gallstone and ensure the continuity of the gastrointestinal tract in high-risk patients. It has been stated in the literature that two-stage surgical treatment is more appropriate in such high-risk patients [3].

## Case presentation

An 83-year-old female patient, with hypertension, applied to an external health center with intermittent right upper quadrant pain two weeks ago. Before admission to the hospital, she had occasional episodes of abdominal pain. While the patient's blood tests revealed high amylase and lipase levels (amylase: 790 U/L, lipase: 810 U/L), the magnetic resonance cholangiopancreatography (MRCP) report described dilatation and a proximal filling defect of the common bile duct. The appearance of the pancreas was found to be normal. Therefore, endoscopic retrograde cholangiopancreatography (ERCP) was performed in the external health center. The patient underwent sphincterotomy and biliary sludge removal during ERCP. Information during the process could not be accessed because ERCP was performed at an external health center. The patient was discharged without any additional procedure or intervention.

The patient, who had complaints of nausea and bilious vomiting one week after discharge, applied to the emergency department of our hospital because of progressive deterioration in oral intake, decreased urine output, shortness of breath, and deterioration in her general condition. She had no additional disease or history of surgery, except for known hypertension. Further history taking also revealed that the last stool output was three days ago. Spontaneous gas discharge was present.

The initial physical examination findings in the emergency room were tachycardia, tachypnea, blurred consciousness, and hypotension. She had no fever and her oxygen saturation in room air was 88%. The patient was monitored. A urinary foley catheter was placed and the patient was observed to be anuric. A nasogastric tube was placed and 500 cc of bile content was aspirated. There was no sign of free air under the diaphragm and no air-fluid level in the posteroanterior (PA) erect abdominal radiograph. Her laboratory test results were as follows: aspartate aminotransferase (AST): 102 U/L, alanine aminotransferase (ALT): 53 U/L, gamma-glutamyl transferase (GGT): 77 U/L, alkaline phosphatase (ALP): 157 U/L, amylase: 159 U/L, lipase: 82 U/L, total bilirubin: 0.81 mg/ dL, direct bilirubin: 0.33 mg/dL, C-reactive protein (CRP): 152 mg/L, creatinine: 6.81 mg/dL, urea: 88.6 mg/dL, sodium (Na): 139 mmol/L, potassium (K): 3.94 mmol/L, calcium (Ca): 10 mg/ dL, pH: 7.28, hemoglobin (Hb): 18.4 g/dL, WBC: 33.93 103/uL, platelet (thrombocyte) count (plt): 462.000 uL. The physical examination revealed no abdominal distension. The bowel sounds were hypoactive, and there was no metallic tinkling sound. There was tenderness in the epigastric region, and Murphy's sign was negative.

**Figure 1:**
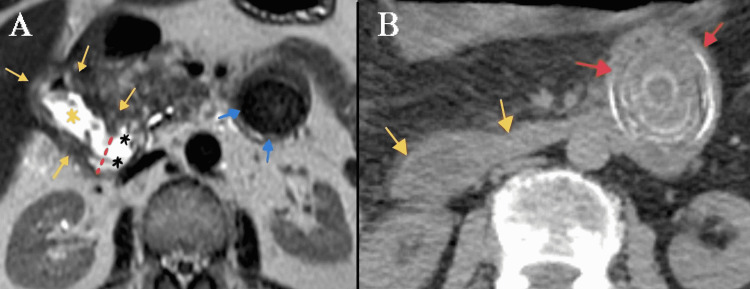
(A) Axial T2-weighted MRI section: Prominently thickened gallbladder wall (yellow arrows) and gallbladder lumen (yellow asterisk) are shown. Cholecystoduodenal fistula (red dashed line) connects gallbladder lumen to duodenal lumen (black asterisks). Giant gallbladder stone (blue arrows) in the proximal jejunum can also be seen; (B) Axial non-contrast CT image section: Duodenal segments (yellow arrows) distal to cholecystoduodenal fistula and giant gallbladder stone (red arrows) in the proximal jejunum are seen.

Vascular access was established and resuscitation was performed with IV isotonic crystalloid fluid. A non-enhanced abdominal computed tomography (CT) performed afterward demonstrated that there was a round-shaped, calcified structure located in the proximal jejunal loop, just distal to the site of Treitz. Additional abdominal magnetic resonance imaging (MRI) was performed in order to evaluate the gallbladder and biliary system. Both MRI and CT confirmed the diagnosis of cholecystoduodenal fistula secondary to gallstone (Figure 1).

After the metabolic resuscitation and initial examinations, emergency surgical exploration was planned for the patient to solve the underlying pathology that caused all this metabolic destruction. The intra-arterial blood pressure of the patient was monitored. The patient was hypotensive and tachycardic before the anesthesia induction. Her operation room entrance Glasgow Coma Score (GCS) was 9. Inotrope infusion was started and 50 mg fentanyl 100 mg ketamine and 50 mg rocuronium were used for induction of anesthesia. After the orotracheal intubation and just before the first incision, the patient developed cardiac arrest. Since the patient was already in the operating room, effective cardiopulmonary resuscitation was started immediately. A total of 2 mg of adrenaline was administered intravenously, one mg at three-minute intervals. Later, normal sinus rhythm was seen. IV crystalloid fluid therapy was continued. She responded to two minutes of effective cardiopulmonary resuscitation and the surgical operation was started immediately after.

**Figure 2:**
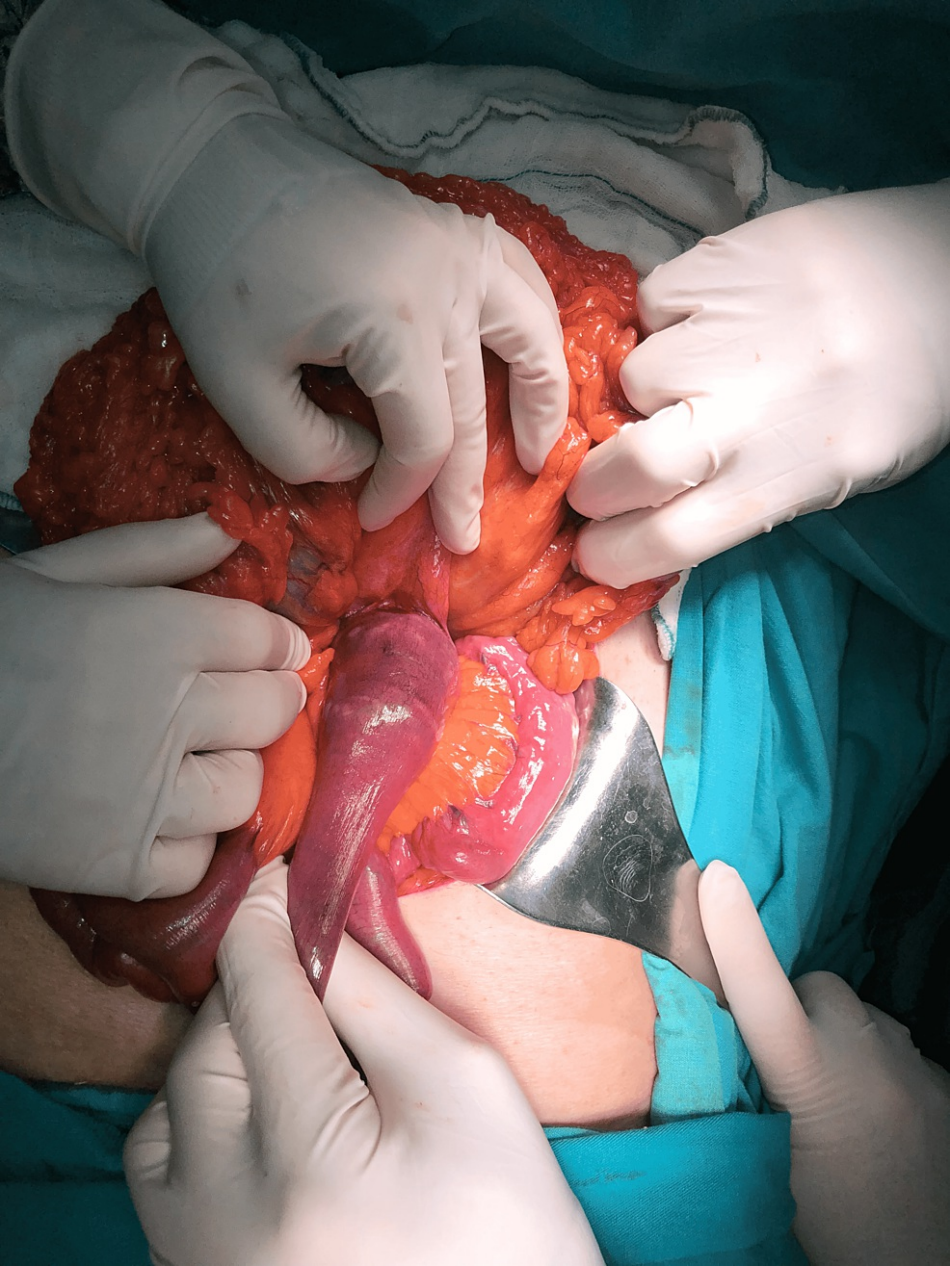
Gallstone completely obstructing the lumen at the level of the ligament of Treitz. Purple-dark appearance of the obstructed intestinal segment, due to decreased blood circulation, can clearly be seen.

The explorative laparotomy was performed with a mini-median incision above and below the umbilicus. No evidence of intra-abdominal free fluid or perforation was found. During the exploration of the intestinal loops, a giant gallstone was palpated at the level of the ligament of Treitz. The intestinal lumen was completely obstructed by the giant gallstone and the intestinal wall circulation was partially disrupted by the compression effect of that (Figure 2).

After further abdominal exploration, dense adhesions of the liver, gallbladder, and duodenal fistula site were seen. Hence, no additional intervention was planned considering intraoperative abdominal findings and the general unstable condition of the patient. No other pathology was detected during the exploration of the abdomen.

**Figure 3:**
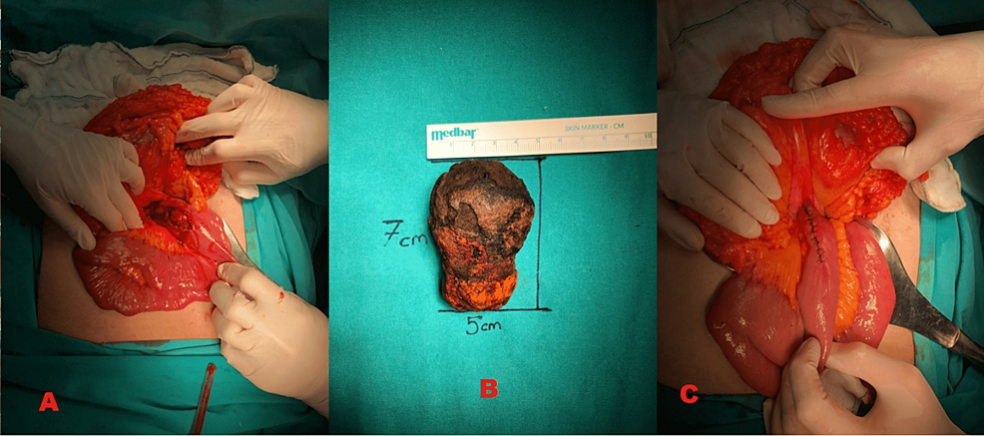
(A) Enterotomy; (B) Removed gallstone; (C) Closure of enterotomy.

Since the gallstone was localized at the level of the ligament of Treitz and there was no suitable area more proximally for enterotomy, an enterotomy was performed with a vertical incision of approximately 5 cm, starting at the level of the ligament of Treitz. The gallstone with 5x4x7 cm dimensions was removed (Figure 3).

The vascularization of the proximal jejunal loop returned to normal as the pressure effect of the gallstone on the wall was removed. Enterotomy was sutured with 3/0 Vicryl using the Connell technique. The double-layered repair was completed by interrupted sutures and Lambert sutures using 3/0 silk. The patency and circulation of the proximal jejunal loop were checked. Leakage was checked by giving 1000 cc of isotonic fluid colored with methylene blue through the nasogastric tube. The operation was terminated in approximately 55 minutes and the patient was taken intubated to the adult intensive care unit.

The condition of the patient, whose metabolic resuscitation was provided and the etiological cause was eliminated, quickly recovered in the postoperative period. The patient was extubated at the post-operative sixth hour. Within 24 hours, the patient's inotropic support was also discontinued and she became normotensive. An average daily urine output of less than 500 cc was observed in the intensive care follow-ups. She was placed on hemodialysis because the creatinine level and potassium level continued to increase for three consecutive days and she had acidosis and hyperkalemia on the third day. After hemodialysis, urine output increased, creatinine and electrolyte values returned to normal, and the amount of content from the nasogastric tube gradually decreased. After hemodialysis, daily urine output reached 100 cc/hour. Oral water was given on the third post-operative day. The oral intake of the patient who had spontaneous gas stool discharge in the follow-up was increased gradually. The patient, was transferred to the inpatient clinic from the intensive care unit with a clean incision, full oral tolerance, spontaneous gas stool discharge, and normal urine output, and was discharged on the 10th post-operative day with recovery.

## Discussion

Gallstone ileus is seen in only 0.3-0.5% of patients with cholecystolithiasis. Due to its rarity, there is no large series of gallstone ileus been published in the literature to date [4]. Most of the cases are in the elderly population. Common symptoms are nausea, vomiting, and abdominal pain. On physical examination, there may be abdominal tenderness and distention; however, there is no obvious specific finding. [5]

The Rigler triad (pneumobilia, presence of abnormal gallstones, and intestinal obstruction) can be seen at a rate of 40-50% on plain abdominal x-ray [6]. All the three signs of Rigler's triad can be seen together only in 15% of the cases on plain abdominal x-rays [7]. Therefore, the use of plain abdominal radiography in the diagnosis is limited. It has been reported in the literature that abdomen CT has a sensitivity of 93% in the diagnosis of gallstone ileus and is the first test to be used for rapid diagnosis [8]. The abdomen CT findings of our patient revealed pneumobilia and an abnormally located gallstone in the proximal jejunum. Since the site of the gall stone is the proximal part of the intestinal system, there was no evident radiological sign of the ileus. CT imaging findings and the patient’s general condition accelerated the surgical decision. Bouveret's syndrome is a disease that explains the cholecystointestinal fistula related to the gastric outlet and duodenum. In our patient, since the gallstone was located in the most proximal part of the jejunum, it was incompatible with Bouveret's syndrome, which is very rare in the literature [9].

Vomiting and dehydration due to intestinal obstruction caused acute renal failure and general condition deterioration in our patient at the time of admission. Despite the emergency intervention, the patient had a cardiac arrest in the operation room due to her advanced age and added comorbidities. Since the patient underwent cardiopulmonary resuscitation and a rapidly performed emergency surgery, it was necessary to postpone the definitive surgery of the fistula site to a second-line surgery. The accurate evaluation of clinical, radiological, and laboratory tests in a multidisciplinary manner is of great importance in not missing the diagnosis.

Our diagnosis and treatment period also made us reevaluate the previous diagnostic and therapeutic procedures that took place in the external health center. None of the records of the previous hospitalization period were presenting any data about fistula or giant gallstone. There was no specific information about an abnormal gallbladder and this evidently indicates diagnostic incompetency. Furthermore, the patient’s complaints and symptoms were still ongoing after ERCP and discharge. Hence, it is highly possible that the patient had symptoms due to cholecystoduodenal fistula from the beginning. Since we only have the written recorded data about the previous hospitalization period, the only assumption that we can generate is that the diagnosis was possibly wrong and overlooked. We may also assume that there is causality between the complication, acute renal failure, that we confronted during the treatment period, and the long-standing period of the symptoms due to misdiagnosis.

## Conclusions

Although gallstone ileus is rare, it has been observed to improve dramatically with early surgical intervention. The proper evaluation of the advanced radiological findings is crucial. It should be noted that the first step of diagnosis and treatment will be "clinical suspicion" and surgery should not be delayed in patients with impaired general condition.
